# Investigating oral microbiome profiles in patients with cleft lip and palate compared with the healthy control

**DOI:** 10.1186/s12903-024-04387-3

**Published:** 2024-05-28

**Authors:** Wenxiu Jiang, Zixin Yan, Zhenwei Chen, Lanxin Gu, Han Bao, Ye Cao, Luwei Liu, Bin Yan

**Affiliations:** 1 Department of Orthodontics, The Affiliated Stomatological Hospital of Nanjing Medical University, Nanjing, 210029 China; 2 State Key Laboratory Cultivation Base of Research, Prevention and Treatment for Oral Diseases, Nanjing Medical University, Nanjing, 210029 China; 3 Jiangsu Province Engineering Research Center of Stomatological Translational Medicine, Nanjing Medical University, Nanjing, 210029 China; 4 Department of Prosthodontics, National Center of Stomatology & National Clinical Research Center for Oral Diseases & National Engineering Research Center of Oral Biomaterials and Digital Medical Devices & Beijing Key Laboratory of Digital Stomatology, Peking University School and Hospital of Stomatology, Haidian District, Beijing, China

**Keywords:** Cleft lip and palate, Oral microbiota, 16S rRNA sequencing, Neisseria abundance

## Abstract

**Background:**

Patients with cleft lip and palate (CLP) have an oronasal communication differed from the closed state in healthy individuals, leading to a unique oral microbiome. This study aimed to determine if variances in the oral microbiota persist among CLP patients who have received treatments for the closure of these fistulas compared to the microbiota of healthy individuals.

**Methods:**

Saliva samples were collected from a cohort comprising 28 CLP patients (CLP group) and 30 healthy controls (HC group). Utilizing 16S rRNA sequencing on the Illumina NovaSeq platform, we conducted a comprehensive analysis of the diversity and composition of the oral microbiota.

**Results:**

The analysis of the microbiota in the saliva samples revealed a total of 23 microbial phyla, 38 classes, 111 orders, 184 families, 327 genera and 612 species. The alpha diversity with microbial abundance and evenness indicated the significant difference between the CLP and HC groups. Principal coordinate analysis (PCoA) and the ADONIS test further supported the presence of distinct microorganisms between the two groups. The CLP group displayed elevated abundances of *Neisseria*, *Haemophilus*, *Porphyromonas*, and *Granulicatella*, as indicated by LefSe analysis. Conversely, *Rothia*, *Veillonella*, and *Pauljensenia* exhibited significant reductions in abundance in the CLP group. The results of the PICRUSt analysis indicated significant differences in the relative abundance of 25 KEGG pathways within the CLP group. Through Spearman correlation analysis, strong associations between *Rothia*, *Veillonella*, and *Pauljensenia* and 25 functional pathways linked to CLP were identified.

**Conclusion:**

Findings of this study offer a thorough comprehension of the microbiome profiles of CLP patients after the restoration of oronasal structure and are anticipated to present innovative concepts for the treatment of CLP.

**Supplementary Information:**

The online version contains supplementary material available at 10.1186/s12903-024-04387-3.

## Background

The cleft lip and palate (CLP) is a prevalent congenital malformation in the oral and maxillofacial region that significantly impacts oral functions such as chewing, pronunciation, aesthetics, and even psychological well-being [1, 2]. This condition is generally attributed to the failure of lambdoidal junction development or fusion [3], resulting in velopharyngeal insufficiency and an open connection between the oral and nasal cavities [4]. Abnormal oral structural conditions can lead to dysbiosis of the oral microbiota, resulting in local oral diseases such as dental caries, gingivitis, and periodontal disease [5]. In addition, it has been demonstrated that oral infections, specifically periodontitis, have a significant impact on the pathogenesis and development of numerous systemic illnesses, including cardiovascular disease, bacterial pneumonia, and diabetes mellitus [5].

Previous studies have found differences in the cariogenic and pathogenic bacteria between individuals with CLP and those in the normal population [6–9]. However, these studies typically depended on conventional microbial culture or DNA-DNA hybridization methods which offered restricted sensitivity and biased selectivity. These methods solely target specific bacteria instead of providing a thorough analysis of the overall microbial community [10]. High-throughput sequencing has enabled several studies to reveal distinct differences in the overall oral microbiome composition between CLP individuals and healthy individuals [11–14]. Zhou et al. discovered that the salivary microbiota of CLP individuals significantly differed from that of healthy individuals utilizing 454-pyrosequencing technology [11]. The research is limited due to the small number of people and samples, and more relevant research needs to be carried out. CLP subjects in prior studies retained oronasal fistulas, exhibiting structural disparities in their oral cavities when compared to healthy populations. However, it remains unknown whether differences in the oral microbiota persist between healthy and CLP individuals without oronasal fistulas after therapies.

In this study, we conducted 16S ribosomal RNA (rRNA) sequencing on an Illumina NovaSeq platform to compare the salivary microbiota between the CLP who have undergone treatments for oronasal fistula closure and healthy individuals.

## Methods

### Subject recruitment and saliva collection

This cross-sectional study recruited participants with nonsyndromic CLP without oronasal fistulas following treatment and healthy controls from the Affiliated Stomatological Hospital of Nanjing Medical University. Approval for the study protocol was granted by the Ethics Committee of the Affiliated Stomatological Hospital at Nanjing Medical University (PJ2022-033-01). The parents or grandparents of these participants were fully informed about the study objectives and provided the written informed consent, as needed by the Ethics Committee.

We recruited patients with CLP (CLP group) aged 8–22 years (mean age 13.2 ± 4.8 years) and healthy controls (HC group) aged 8–24 years (mean age 14.3 ± 4.7 years). Individuals with gingivitis, periodontitis, or systemic diseases as well as those who had consumed antibiotics or probiotics within the 2 weeks preceding sampling, were excused from participating. The detailed information of the participants was shown in Table 1. Unstimulated saliva samples were collected using sterile tubes from 58 individuals in the morning between 8:00–9:00, following an 8-hour fasting period without brushing their teeth. A trained dentist supervised the collection of unstimulated saliva from each participant, which was subsequently transferred to 1.5-mL sterile microcentrifuge tubes. The samples were frozen at -80 °C for future use.

### DNA extraction and 16S rRNA gene sequencing

Following the manufacturer’s instructions, DNA was extracted from various samples using the cetyl trimethyl ammonium bromide (CTAB) method (details see Supplementary Method) [15]. PCR was used to amplify the hypervariable regions of the bacterial 16S rRNA gene known as V3-V4. The primer sets 341 F (5′-CCTACGGGNGGCWGCAG-3′) and 805R (5′-GACTACHVGGGTATCTAATCC-3′) were employed for this purpose [16]. The bacterial 16S rRNA genes were amplified using a PCR procedure that comprised an initial 30 s denaturation step at 98 °C, 32 cycles of 10 s denaturation at 98 °C, 30 s annealing at 54 °C, and 45 s extension at 72 °C, and a final 10 min extension at 72 °C. During PCR, ultrapure water was used as a negative control to exclude the interference of false positives. Confirmation of the amplified PCR products was conducted through agarose gel electrophoresis, utilizing a 2% agarose gel. Following confirmation, the PCR products was purified utilizing AMPure XT beads (Beckman Coulter Genomics, Danvers, MA, USA). Quantification of the purified PCR products was then performed utilizing Qubit (Invitrogen, USA). In order to facilitate the sequencing process, we evaluated the quantities and dimensions of the amplicon pools using a Library Quantification Kit for Illumina (Kapa Biosciences, Woburn, MA, USA) and an Agilent 2100 Bioanalyzer (Agilent, USA), respectively. Subsequently, the NovaSeq PE250 platform was utilized to carry out the sequencing of the libraries.

### Data processing and bioinformatic analysis

Sequencing was performed on an Illumina NovaSeq platform following the manufacturer’s guidelines. The distinct barcodes were utilized to assign the paired-end reads to their corresponding samples. The paired-end reads were merged using FLASH (version 1.2.8) [17] after eliminating the barcode and primer sequence, applying parameters with a minimum overlap threshold of 10, a maximum threshold of 100, and a maximum allowed mismatch ratio of 0.25 for accurate merging. To ensure the production of high-quality clean tags, the raw reads underwent quality filtering with fqtrim (version 0.94) under specific filtering conditions. Subsequently, the removal of chimeric sequences was performed using Vsearch software (version 2.3.4) [18]. By implementing DADA2 [19] for dereplication with the truncation length set at 400, a feature table and feature sequence were obtained. Normalization to the same randomly selected sequences allowed for the calculation of alpha diversity and beta diversity. The normalization of feature abundance was carried out by using the relative abundance of each sample based on the Greengenes2 reference database (version 2022.10) [20].

Bioinformatics analysis was conducted using QIIME2 (version 2023.5) [21]. Alpha diversity analysis was conducted to assess species diversity within each sample through 6 indices, including Chao1, observed species, Pielou evenness index, abundance-based coverage estimator (ACE), Simpson index and Shannon–Wiener index. Beta diversity analysis was performed using principal coordinate analysis (PCoA) based on weighted UniFrac distances at the ASV level. We employed the ADONIS [22] test to determine the significance of beta diversity differences. For biomarker discovery, we utilized linear discriminant analysis (LDA) effect size analysis (LEfSe) with a cut-off of 4.0 on the logarithmic LDA score [23], on the online platform of Wekemo Bioincloud (https://www.bioincloud.tech). Additionally, we employed the online platform of Wekemo Bioincloud for the Phylogenetic Investigation of Communities by Reconstruction of Unobserved States (PICRUSt) [24]. This allowed us to predict microbial functions by annotating the gene catalog based on the Kyoto Encyclopedia of Genes and Genomes (KEGG) modules [25]. Lastly, we conducted Spearman correlation analysis using the R package to examine the relationship between different genera and KEGG pathways. R version 4.3.1, vegan version 2.6-4, ggplot2 version 3.4.4, corrplot version 0.92, psych version 2.3.9 and phyloseq version 1.44.0 packages [26] were utilized for the above statistical analysis and generation of diagrams.

### Statistical analysis

We utilized chi-square tests and Student’s t tests to compare the demographic data, analyzing categorical and continuous variables that follow a normal distribution, respectively. The alpha diversity, following its assessment for normal distribution, was compared with Student’s tests. To evaluate the significant separation of clusters based on the weighted UniFrac distance matrix, we conducted ADONIS. Statistical significance was determined using a *P* value threshold of less than 0.05. In order to compare the relative abundances of ASVs between groups, we performed LEfSe. Additionally, we used Welch’s t test to assess differences in the relative abundance of KEGG pathways between the two groups. To minimize the likelihood of false positives, we considered Bonferroni-corrected *P* values below 0.01 to indicate statistical significance.

## Results

### Characteristics of subjects

Based on the study criteria, two patients in the CLP group were excluded, and a total of 58 subjects were identified. The clinical demographics of the subjects were presented in Table 1, including 28 CLP patients (7 men, 21 women) and 30 healthy controls (10 men, 20 women). The mean age of the CLP group was 13.2 years, with 53.6% in the mixed dentition stage and 46.4% in the permanent dentition stage. The HCs had a mean age of 14.3 years, with 33.3% in the mixed dentition stage and 66.7% in the permanent dentition stage. No significant differences in sex, dentition or age were found between the groups (Table 1).

**Table 1:** Basic characteristics of the participants

	CLP (*N* = 28)	HC (*N* = 30)	*P* value
**Gender**			
Male	7 (25.0%)	10 (33.3%)	0.683
Female	21 (75.0%)	20 (66.7%)	
Age	13.2 (4.8)	14.3 (4.7)	0.404
**Dentition**			
Mixed dentition	15 (53.6%)	10 (33.3%)	0.197
Permanent dentition	13 (46.4%)	20 (66.7%)	

*Note* Mean (standard deviation) was used to present continuous variables, while the number of individuals (percentage) was used to present other categorical variables. CLP: cleft lip and palate; HC: healthy control

### Microbial profiles of saliva samples

**Fig. 1:**
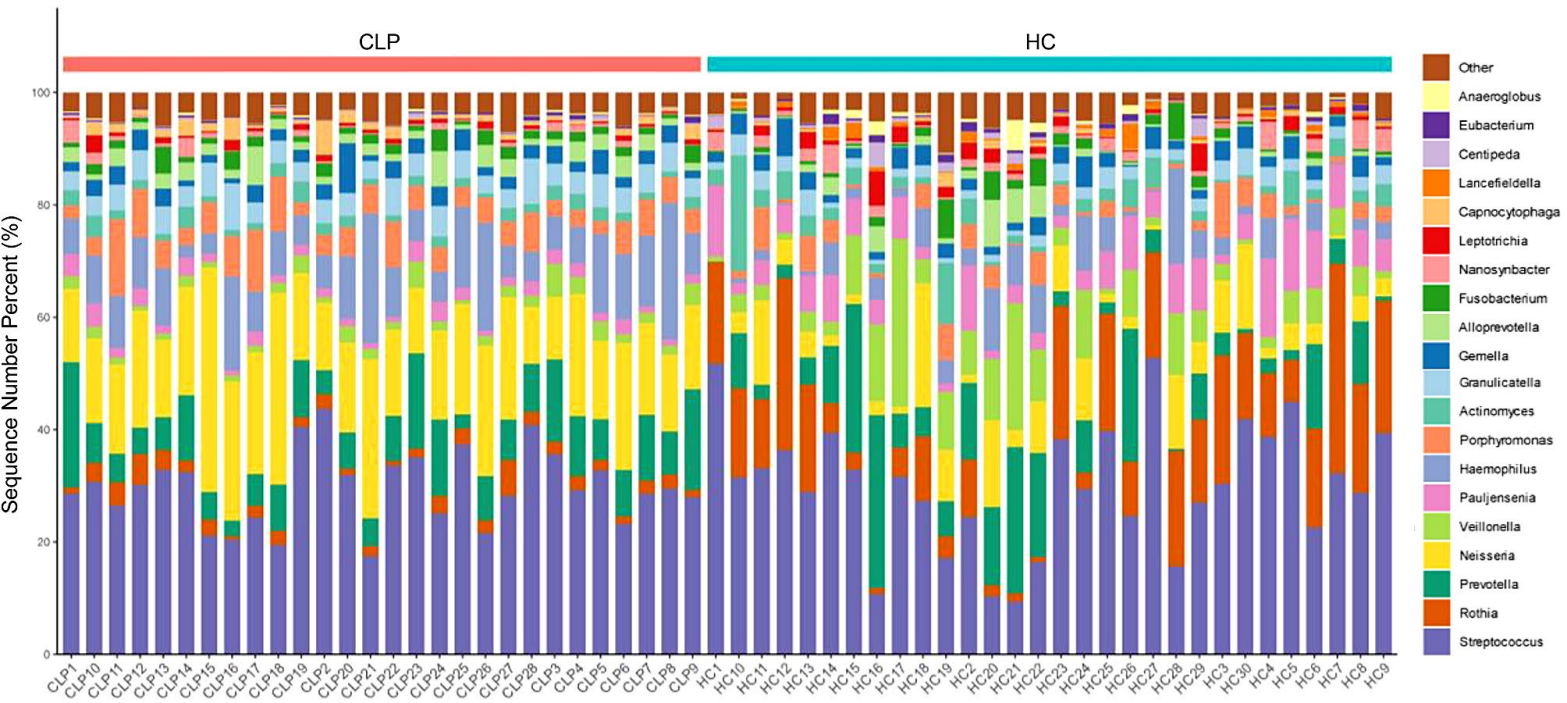
Microbiological profiles at the genus level in the CLP and HC groups. The bar graphs illustrate the mean relative abundances of the top 20 genera identified in the study cohorts. CLP: cleft lip and palate; HC: healthy control

From the 58 saliva profile data, 2,581,390 final reads were generated after filtering, with a mean of 44,507 (range 5,309–78,188) reads per sample. We ultimately detected 2384 amplicon sequence variants (ASVs). Microbiota analysis revealed 23 microbial phyla, 38 classes, 111 orders, 184 families, 327 genera and 612 species in the saliva samples. The oral microbial composition of both groups exhibited a dominance of the following genera with a relative abundance exceeding 5% (CLP; HC): *Streptococcus* (29.61%; 30.27%), *Neisseria* (18.62%; 5.99%), *Prevotella* (8.82%; 8.93%) in Table S1. The results for the top 20 genera and species were respectively shown in Fig. 1 and Fig. S1.

**Fig. 2:**
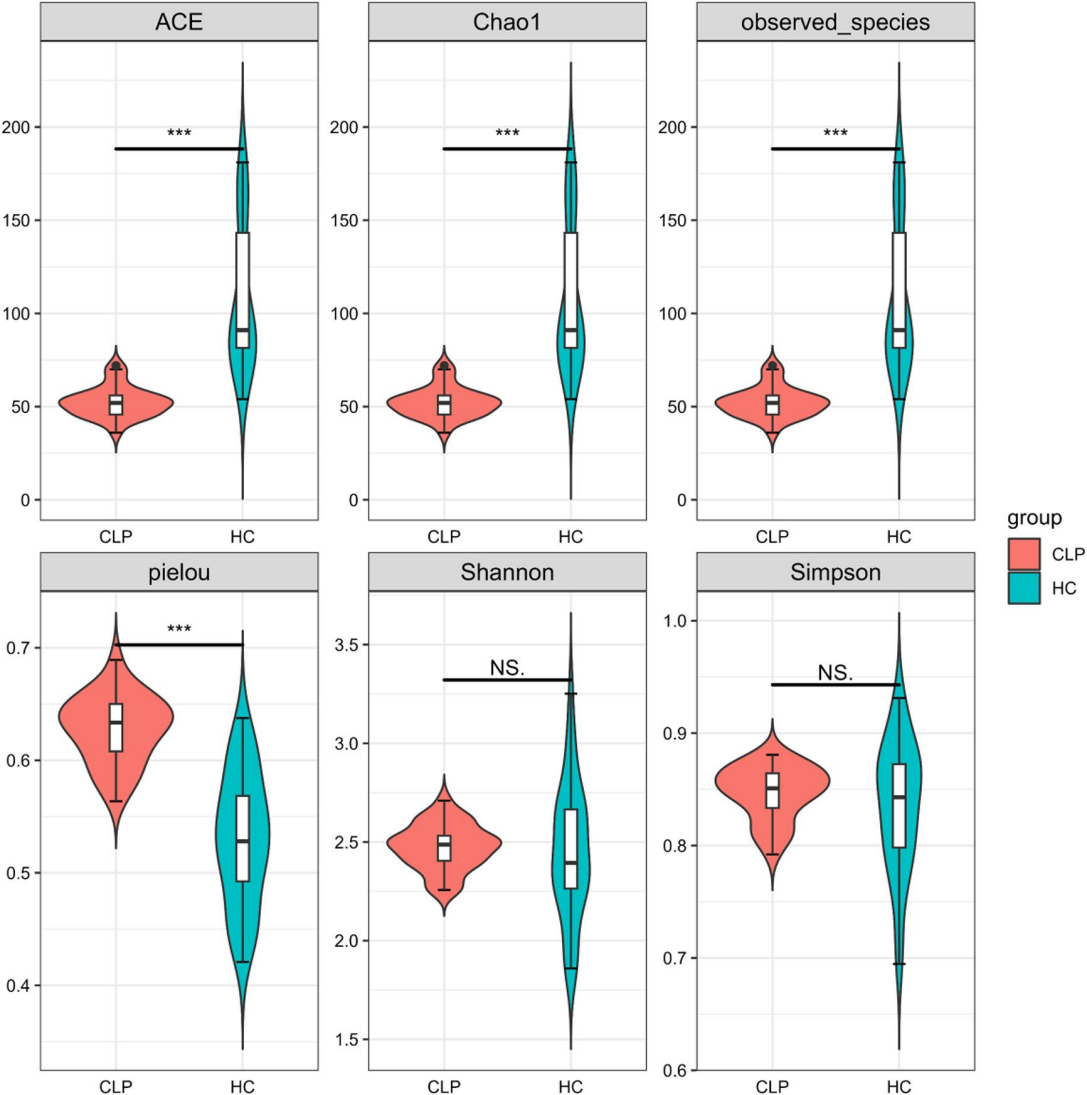
The alpha diversity of the saliva microbiome between the CLP and HC groups. The indices are Abundance-based Coverage Estimator (ACE), Chao1, observed species, Simpson Index (Simpson), Shannon-Wiener index (Shannon) and Pielou evenness index (Pielou) in the order. CLP: cleft lip and palate; HC: healthy controls; NS. No significance; *** *P* < 0.001

Species richness (number of ASVs) or evenness (relative abundance of ASVs) in a single sample determines the alpha diversity of an ecosystem. There was no significant variation observed in the overall species diversity, assessed through the Shannon-Wiener and Simpson indices (*P* > 0.05; Fig. 2), between the CLP and HC groups. However, within the CLP group, there was a possibility of a more evenly distributed microbial community with a greater Pielou evenness index (*P* < 0.001; Fig. 2). Conversely, in the HC group, even though there were more microbial species (ACE, Chao1, and observed species were larger, *P* < 0.001; Fig. 2), they might not be distributed evenly. These results suggested that microbial communities in the CLP might have unique ecological traits and structural differences.

**Fig. 3:**
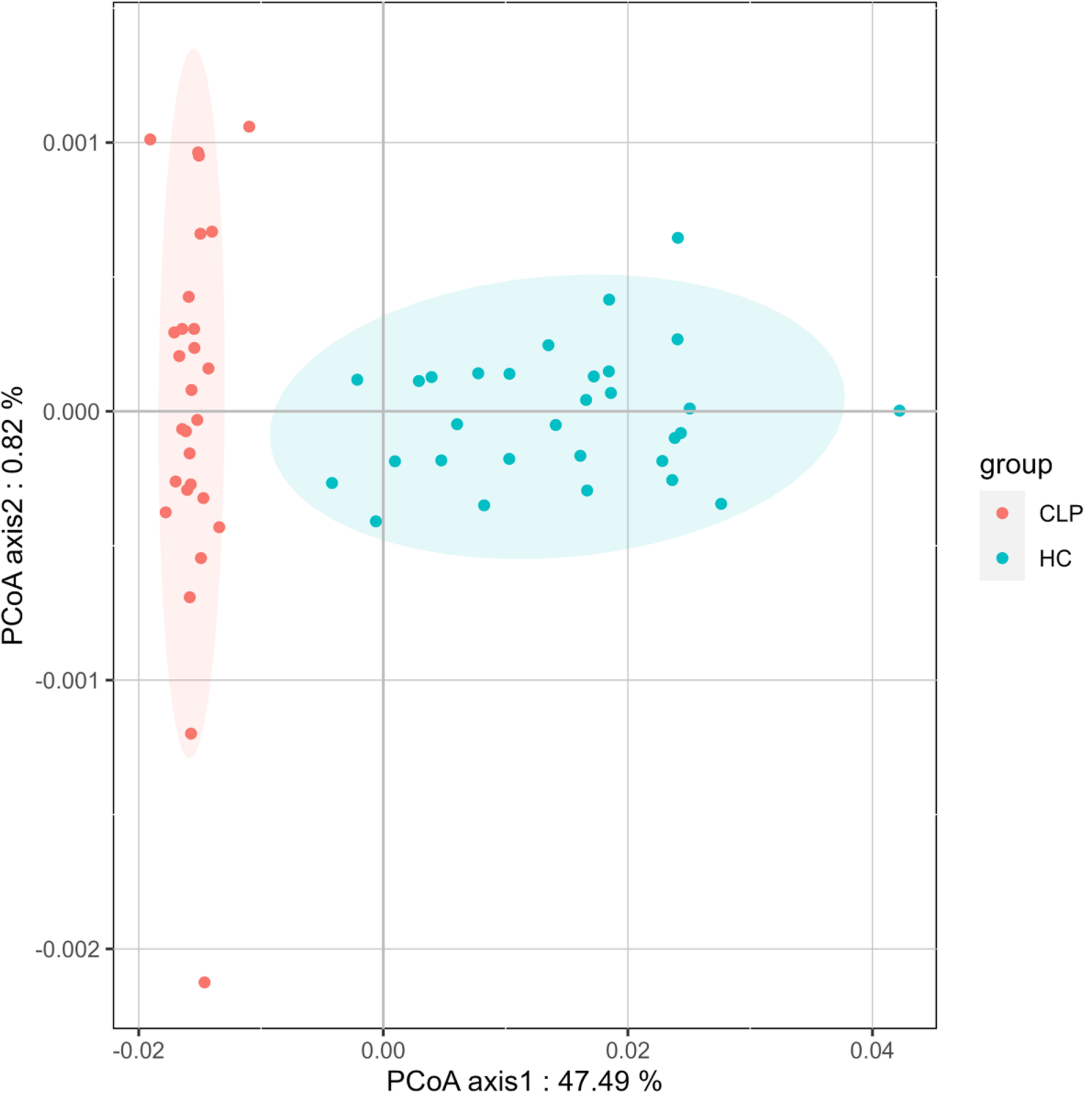
Beta diversity of the oral microbiota of the CLP and HC groups. PCoA plots indicated a marked distinction between the CLP and HC groups based on the weighted UniFrac distance. CLP: cleft lip and palate; HC: healthy control

Weighted UniFrac distance measurements were employed to compare the variation in the composition of salivary bacterial communities between the CLP and HC groups. PCoA plots showed distinct clusters representing the CLP and HC groups, indicating a significant difference in their salivary microbial communities (Fig. 3). This significant difference was further confirmed by ADONIS (*P* = 0.001; R^2^ = 0.237).

### Variations in each study group’s oral microbiota

**Fig. 4:**
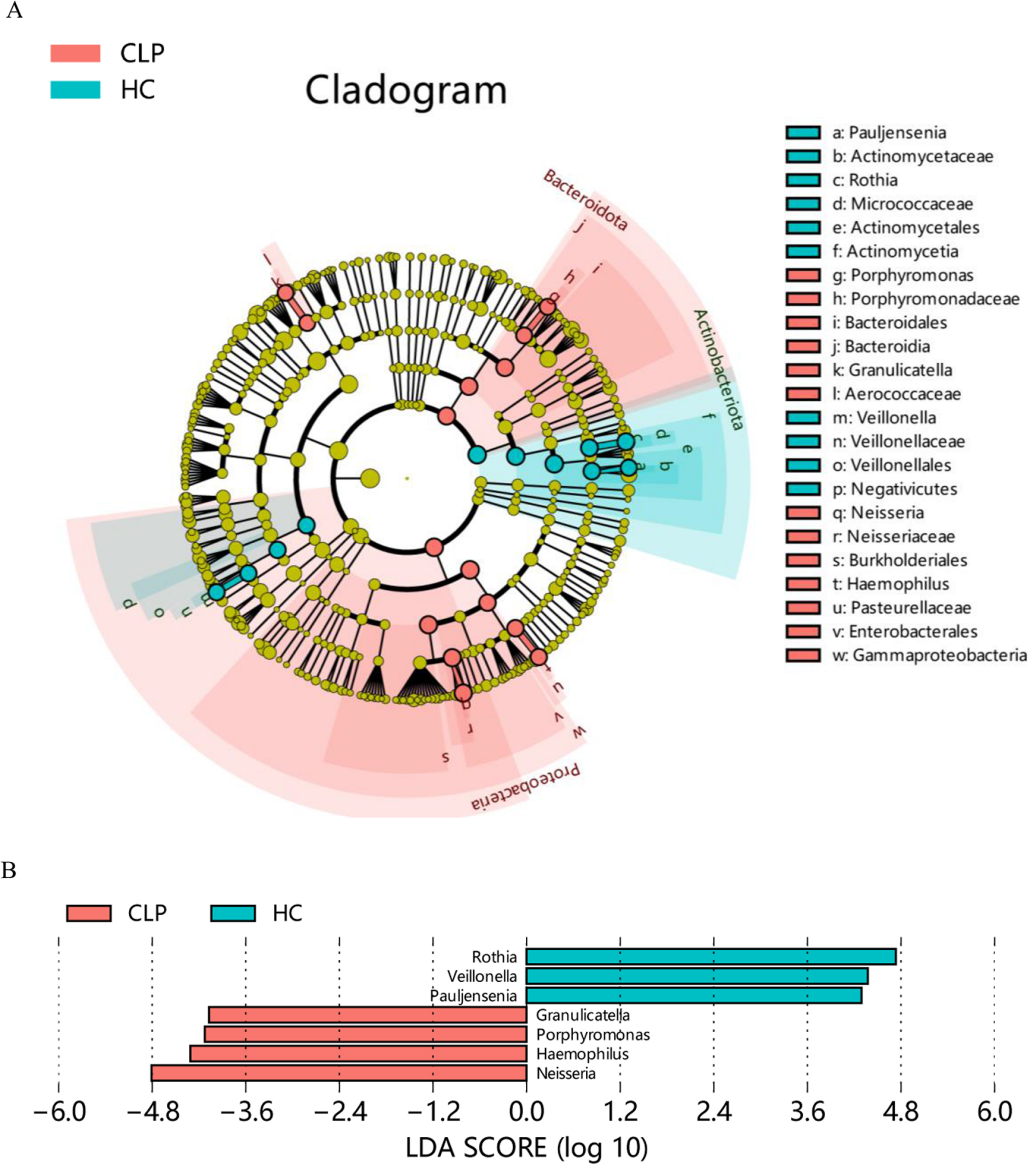
Differential abundance analysis of salivary microbiota constituents between study groups based on the LEfSe method. **A** Phylogenetic tree presenting the hierarchical relationships among various taxa, ranging from the phylum level to the genus level. **B** Marker microbes in the CLP and HC groups with LDA score (log10) > 4. The difference was more pronounced the higher the LAD. CLP: cleft lip and palate; HC: healthy control

To investigate the impact of CLP deformity on the oral bacterial composition, we performed a comparative investigation to analyze the relative abundance of microbial communities in the saliva of two groups: CLP and HC. Using LEfSe, we identified 7 genera that were notably different between the study groups, with LDA scores greater than 4. The HC group exhibited significant increases in the abundances of *Rothia*, *Veillonella*, and *Pauljensenia*, while the CLP group had greater abundances of *Neisseria*, *Haemophilus*, *Porphyromonas*, and *Granulicatella* (Fig. 4B). These genera could serve as markers for the CLP group. Based on the hierarchical relationships, the depicted phylogenetic tree illustrated the marker taxa ranging from the phylum to the genus levels (Fig. 4A). Within the CLP group, two marker branches were observed: *Bacteroidota-Bacteroidia-Bacteroidales-Porphyromonadaceae-Porphyromonas* and *Proteobacteria-Gammaproteobacteria-Enterobacterales-Pasteurellaceae-Haemophilus*. On the other hand, the HC group had two marker branches: *Actinobacteriota-Actinomycetia-Actinomycetales-Micrococcaceae-Rothia* and *Actinobacteriota-Actinomycetia-Actinomycetales-Actinomycetaceae-Pauljensenia*. The enrichment of cariogenic bacteria and periodontal pathogens in individuals with CLP may indicate a correlation with the higher incidence of dental caries and periodontal diseases associated with CLP [27–29].

### Potential function of the oral microbiome in CLP patients

**Fig. 5:**
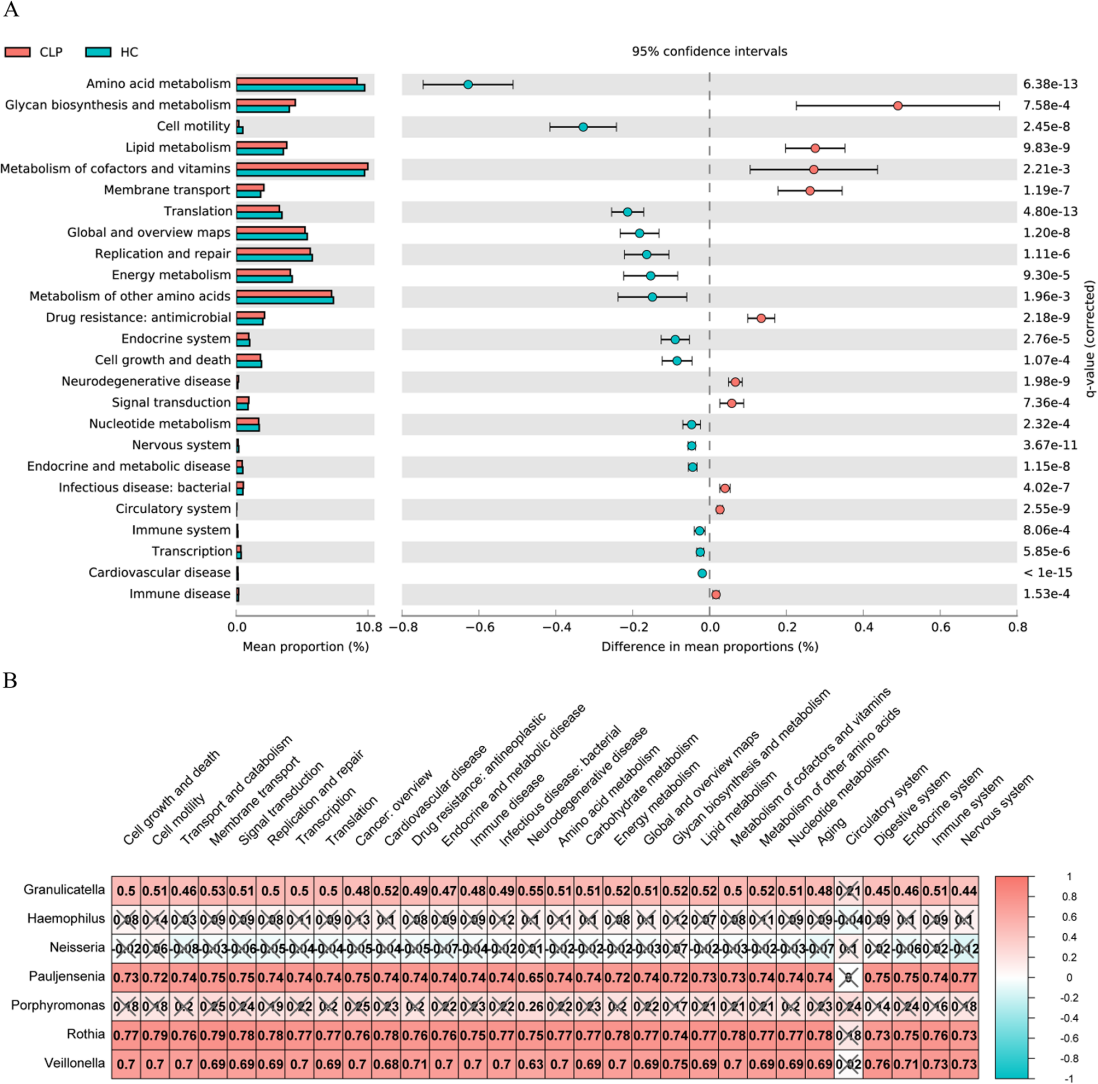
Functional prediction by PICRUSt 2. **A** Twenty-five KEGG pathways with significant differences across the groups according to Welch’s t test (*P* < 0.05, corrected by Benjamini–Hochberg). **B** Seven genera were identified as differentially correlated with pathways through Spearman correlation analysis. Positive correlations between genera and pathways are represented by red, while negative correlations are represented by blue. The numbers indicate the Spearman correlation coefficient. CLP: cleft lip and palate; HC: healthy control

Functional pathways associated with the salivary microbiota of CLP patients were predicted through PICRUSt analysis of the ASV table. A total of twenty-five functional pathways were found to be differentially abundant between CLP and HC groups (*P* < 0.05, Welch’s t test). Among these pathways, 15 (amino acid metabolism, cell motility, translation, immune system, energy metabolism, endocrine system, replication and repair, global and overview maps, nervous system, metabolism of other amino acids, cell growth and death, nucleotide metabolism, endocrine and metabolic disease, transcription and cardiovascular disease) were downregulated, while ten pathways (lipid metabolism, membrane transport, drug resistance: antimicrobial, glycan biosynthesis and metabolism, neurodegenerative disease, signal transduction, metabolism of cofactors and vitamins, infectious disease: bacterial, circulatory system and immune disease) were upregulated in the CLP group (Fig. 5A). The Spearman correlation analysis was conducted on the 25 distinct functional pathways and 7 unique bacterial genera (Fig. 5B). *Rothia*, *Veillonella*, and *Pauljensenia*, which were the taxa whose abundance decreased in the CLP group, exhibited positive correlations with most KEGG pathways, albeit with similar strengths. On the other hand, *Neisseria*, *Haemophilus*, and *Porphyromonas*, which were the genera that increased in abundance in the CLP group, did not show any correlation with the functional pathways, except for *Granulicatella*, which exhibited a weak correlation. Furthermore, it is worth noting that the circulatory system pathway did not correlate with any of the seven differential genera. Changes in the differential KEGG metabolic pathways associated with divergent bacterial genera suggest that intracellular homeostasis, energy production, and tissue development may be compromised, thereby influencing the high incidence of inflammatory diseases in CLP individuals [30].

## Discussion

The potential spatial variability in geomorphic and environmental characteristics influences the structure and functional spatial pattern of the bacterial community. Bacteria from differing ecological sites, such as the nasal passages, oral cavity, and pharynx, communicate with each other or spread throughout other remote regions within the organism in cases of CLP. The connection between the oral and nasal cavities in CLP patients can have an impact on the oral microecological environment. This can potentially disrupt the oral microbiome and increase the susceptibility of CLP patients to certain diseases [8]. This study provided an analysis of the salivary microbiome in individuals with and without CLP following the restoration of oronasal structure by employing 16S rRNA sequencing on the Illumina NovaSeq platform. Our analysis took an overall microbial community approach and explored the characteristics of the oral microbiota, as well as potential changes in functional pathways associated with CLP.

Statistical differences were observed in alpha diversity between the CLP and HC groups across datasets using standard estimates, such as ACE, Chao1, observed species, and pielou. These findings have been previously reported in other studies [31]. The low diversity of salivary microbiota in the CLP group reduces the stability of the microecological environment, making them more susceptible to diseases like dental caries [32]. However, a separate investigation uncovered a lack of notable disparity in alpha diversity when comparing the CLP and HC cohorts [11]. The discrepancy in the study results could be attributed to the limited number of participants and the classification of participants into various groups. This could have resulted in a decreased statistical power, hindering the accuracy and reliability of the findings. We employed ADONIS testing to evaluate beta diversity and found significant associations between groups, as indicated by the weighted UniFrac distance. These findings align with Funahashi et al.‘s study [33]. Previous research has indicated that CLP patients experience transient changes in their microbial community shortly after surgical treatment, but the oral microbiota tends to revert to its pre-surgical state over time [34]. This suggests that the significant differences in the oral microbiota between patients with repaired oronasal fistulas in CLP and healthy individuals may stem from the long-standing oronasal communication in CLP patients, which has altered their inherent oral microbiota, leading to the establishment of an unique oral microecology [35]. Even subsequent treatments that modify the oral structure to resemble that of healthy individuals may not easily alter the established intrinsic oral microbial community.

We further compared the oral microbial composition between the CLP group and the HC group based on LEfSe. The abundance of oral pathogenic bacteria, including *Neisseria*, *Haemophilus*, *Porphyromonas*, and *Granulicatella*, was found to be increased in the CLP group. Prior research has established a strong association between *Neisseria* and the occurrence and development of cavities, as it produces acid that reduces the pH of the mouth and leads to enamel demineralization [36]. The notable prevalence of *Neisseria* genus in the CLP group is consistent with previous research findings [37], underscoring its potential as a caries indicator [27]. The *Haemophilus* genus, which consists of opportunistic pathogens, is often found in infants before undergoing the first stage of repair of the soft palate [38]. This is particularly relevant as *Haemophilus* genus is a common pathogen associated with otitis media, a frequent complication in CLP patients [39]. *Porphyromonas*, known for its association with periodontal diseases [28], could play a key role in the development of oral health issues in CLP individuals. Variations in *Porphyromonas* abundance may disrupt microbial equilibrium, increasing the risk of periodontal infections. Additionally, *Granulicatella*, a significant contributor to plaque biofilm formation, may have a crucial role in plaque-related conditions like periodontal disease and dental caries in individuals with CLP [40]. These findings imply that the increased abundance of these genera in the CLP group could potentially contribute to the progression of plaque disease or other bacterial infectious diseases, such as otitis media.

In parallel, our analysis also demonstrated a decrease in the abundance of *Rothia*, *Veillonella*, and *Pauljensenia* in the CLP group. *Rothia*, *Veillonella*, and *Pauljensenia* are common oral commensal flora [41]. The decreased abundance of *Rothia*, recognized for its capacity to produce antimicrobial compounds and promote oral health, may suggest a compromised defense mechanism against harmful pathogens in CLP patients [42]. Similarly, *Veillonella* [43] is associated with lactate and short-chain fatty acid metabolism, which contributes to the maintenance of oral pH and overall oral health. This reduction may disrupt microbial equilibrium, potentially affecting the oral environment’s resilience against acid-producing bacteria and, consequently, increasing susceptibility to oral diseases. Moreover, further studies of *Pauljensenia*, a genus recently renamed Actinomyces, are needed to determine its specific functional characteristics in the oral microbiota of CLP patients [44].

To describe the functional characteristics of the CLP microbiome, we profiled the functions of all saliva samples via the KEGG database. The identification of 25 differentially enriched KEGG pathways revealed the functional landscape associated with CLP, with the most prominent differences observed in glycan biosynthesis and metabolism, cell motility, amino acid metabolism, the metabolism of cofactors and vitamins, and lipid metabolism. Notably, within the CLP group, lipid metabolism, glycan biosynthesis and metabolism, and metabolism of cofactors and vitamins were upregulated, while amino acid metabolism and cell motility pathways were downregulated. Spearman correlation analysis further revealed strong associations between the decreased abundances of the *Rothia*, *Veillonella*, and *Pauljensenia* genera and the 25 differentially enriched KEGG pathways. This correlation suggested a potential link between alterations in microbial composition and the observed functional changes in CLP patients. The upregulation of lipid metabolism, metabolism of cofactors and vitamins, as well as glycan biosynthesis and metabolism in the CLP group indicated a shift in functional priorities, potentially reflecting changes in energy utilization, signaling pathways, and biosynthetic processes [45–47]. These alterations may have implications for cell surface structures, extracellular matrix composition, and overall cellular function [48]. Conversely, the downregulation of amino acid metabolism and cell motility pathways in the CLP group suggested potential disruptions in cellular homeostasis, energy production, and tissue development [49]. The correlation observed with certain decreasing genera highlights the complex relationship between the composition and function of the oral microbiota in individuals with CLP. However, no correlation was found between the KEGG pathways and increase in *Neisseria*, *Haemophilus*, or *Porphyromonas* in the CLP group, while a weak correlation was observed between the functional pathways and Granulicatella. These findings suggest that the differences in functional pathways between CLP patients and healthy individuals may be primarily associated with the decrease in commensal bacteria rather than the increase in pathogenic bacteria. Therefore, the control of oral microbes in CLP patients may begin by replenishing commensal flora and adjusting the balance of microbial flora rather than solely focusing on eliminating pathogenic bacteria.

Our research revealed the differences of the oral microbiota and potential metabolic pathways between CLP individuals with oronasal fistula closure and healthy individuals, suggesting that anatomical repair alone might not suffice to reconcile the disparities in the oral microbial ecosystem between CLP individuals and the general population. Nonetheless, our cross-sectional design was unable to capture its temporal dynamics or changes pre- and post-CLP treatment. It remained uncertain whether the specific microbiota identified in CLP individuals correlated with an increased risk of CLP-associated complications, such as a higher incidence of caries and periodontitis. Future research should employ metagenomic or metabolomic sequencing to delve into the functional differences between CLP individuals who have undergone oronasal fistula repair and healthy individuals. Subsequent in vivo and in vitro experiments should be conducted to functionally validate the impact of these differential bacteria on disease outcomes associated with CLP.

## Conclusion

The structure and composition of the salivary microbiota in CLP patients after oronasal fistula closure significantly differed from those of healthy individuals. The presence of *Neisseria*, *Haemophilus*, *Porphyromonas*, and *Granulicatella* was significantly increased in CLP patients, while the abundance of *Rothia*, *Veillonella*, and *Pauljensenia* significantly decreased. The reduced abundance of these bacteria was closely associated with the distinct functional pathways observed in the CLP group. Therefore, managing the oral microbiota in CLP patients after oronasal fistula closure should prioritize enhancing the commensal flora to preserve the oral microbial ecosystem’s equilibrium.

### Electronic supplementary material

Below is the link to the electronic supplementary material.

Supplementary Material 1

## Data Availability

The raw sequences have been deposited in the NCBI Sequence Read Archive under the accession numbers PRJNA1091054 and PRJNA432821. These SRA records will be accessible via the following links: https://www.ncbi.nlm.nih.gov/sra/PRJNA1091054 and https://www.ncbi.nlm.nih.gov/sra/PRJNA432821.

## Abbreviations

CLP: Cleft and palate
HC: Healthy control
16S rRNA: 16S ribosomal RNA
ANOSIM: Analysis of similarities
CTAB: Cetyl trimethyl ammonium bromide
ACE: Abundance-based coverage estimator
LDA: Linear discriminant analysis
KEGG: Kyoto Encyclopedia of Genes and Genomes
ASVs: Amplicon sequence variants
PCoA: Principal coordinate analysis
LEfSe: Linear discriminant analysis of effect size
PICRUSt2: Phylogenetic investigation of communities by reconstruction of unobserved states

## References

[1] Mossey PA, Little J, Munger RG, Dixon MJ, Shaw WC (2009). Cleft lip and palate. Lancet (London England).

[2] Szyszka-Sommerfeld L, Woźniak K, Matthews-Brzozowska T, Kawala B, Mikulewicz M (2017). Electromyographic analysis of superior orbicularis oris muscle function in children surgically treated for unilateral complete cleft lip and palate. J cranio-maxillo-facial Surgery: Official Publication Eur Association Cranio-Maxillo-Facial Surg.

[3] Kurosaka H, Iulianella A, Williams T, Trainor PA (2014). Disrupting hedgehog and WNT signaling interactions promotes cleft lip pathogenesis. J Clin Investig.

[4] Seselgyte R, Bryant D, Demetriou C, Ishida M, Peskett E, Moreno N, Morrogh D, Sell D, Lees M, Farrall M (2019). Disruption of FOXF2 as a likely cause of Absent Uvula in an Egyptian family. J Dent Res.

[5] Wang D, Nambu T, Tanimoto H, Iwata N, Yoshikawa K, Okinaga T, Yamamoto K. Interdental Plaque Microbial Community Changes under in Vitro Violet LED irradiation. Antibiot (Basel Switzerland) 2021, 10(11). 10.3390/antibiotics10111348 PMC8614803 34827286

[6] Ahluwalia M, Brailsford SR, Tarelli E, Gilbert SC, Clark DT, Barnard K, Beighton D (2004). Dental caries, oral hygiene, and oral clearance in children with craniofacial disorders. J Dent Res.

[7] Sundell AL, Ullbro C, Dahlén G, Marcusson A, Twetman S (2018). Salivary microbial profiles in 5-year old children with oral clefts: a comparative study. Eur Archives Pediatr Dentistry: Official J Eur Acad Pediatr Dentistry.

[8] Perdikogianni H, Papaioannou W, Nakou M, Oulis C, Papagiannoulis L (2009). Periodontal and microbiological parameters in children and adolescents with cleft lip and /or palate. Int J Pediatr Dent.

[9] Machorowska-Pieniążek A, Mertas A, Skucha-Nowak M, Tanasiewicz M, Morawiec T. A comparative study of oral microbiota in infants with complete cleft lip and palate or cleft soft palate. Biomed Res Int 2017, 2017:1460243. 10.1155/2017/1460243 PMC5368409 28393073

[10] Sundell AL, Ullbro C, Dahlén G, Marcusson A, Twetman S (2018). Salivary microbial profiles in 5-year old children with oral clefts: a comparative study. Eur Archives Pediatr Dentistry.

[11] Zhou F, Su Z, Li Q, Wang R, Liao Y, Zhang M, Li J. Characterization of bacterial differences Induced by Cleft-Palate-Related Spatial Heterogeneity. Pathogens (Basel Switzerland) 2022, 11(7). 10.3390/pathogens11070771 PMC9323727 35890015

[12] Funahashi K, Shiba T, Watanabe T, Muramoto K, Takeuchi Y, Ogawa T, Izumi Y, Sekizaki T, Nakagawa I, Moriyama K (2019). Functional dysbiosis within dental plaque microbiota in cleft lip and palate patients. Prog Orthodont.

[13] Seidel CL, Strobel K, Weider M, Tschaftari M, Unertl C, Willershausen I, Weber M, Hoerning A, Morhart P, Schneider M (2023). Orofacial clefts alter early life oral microbiome maturation towards higher levels of potentially pathogenic species: a prospective observational study. J oral Microbiol.

[14] Chen IL, Huang F, Li SC, Huang HC (2023). Salivary microbiome and asthma risk in children with orofacial defects. Pediatr Pulmonol.

[15] Chen F, Ye J, Chio C, Liu W, Shi J, Qin W (2020). A simplified quick microbial genomic DNA extraction via freeze-thawing cycles. Mol Biol Rep.

[16] Logue JB, Stedmon CA, Kellerman AM, Nielsen NJ, Andersson AF, Laudon H, Lindström ES, Kritzberg ES (2016). Experimental insights into the importance of aquatic bacterial community composition to the degradation of dissolved organic matter. Isme j.

[17] Magoč T, Salzberg SL (2011). FLASH: fast length adjustment of short reads to improve genome assemblies. Bioinf (Oxford England).

[18] Rognes T, Flouri T, Nichols B, Quince C, Mahé F (2016). VSEARCH: a versatile open source tool for metagenomics. PeerJ.

[19] Callahan BJ, McMurdie PJ, Rosen MJ, Han AW, Johnson AJ, Holmes SP (2016). DADA2: high-resolution sample inference from Illumina amplicon data. Nat Methods.

[20] McDonald D, Jiang Y, Balaban M, Cantrell K, Zhu Q, Gonzalez A, Morton JT, Nicolaou G, Parks DH, Karst SM et al. Greengenes2 unifies microbial data in a single reference tree. Nat Biotechnol 2023. 10.1038/s41587-023-01845-1 PMC10818020 37500913

[21] Bolyen E, Rideout JR, Dillon MR, Bokulich NA, Abnet CC, Al-Ghalith GA, Alexander H, Alm EJ, Arumugam M, Asnicar F (2019). Author correction: reproducible, interactive, scalable and extensible microbiome data science using QIIME 2. Nat Biotechnol.

[22] Anderson MJ, Walsh DC (2013). PERMANOVA, ANOSIM, and the Mantel test in the face of heterogeneous dispersions: what null hypothesis are you testing?. Ecol Monogr.

[23] Segata N, Izard J, Waldron L, Gevers D, Miropolsky L, Garrett WS, Huttenhower C (2011). Metagenomic biomarker discovery and explanation. Genome Biol.

[24] Douglas GM, Maffei VJ, Zaneveld JR, Yurgel SN, Brown JR, Taylor CM, Huttenhower C, Langille MGI (2020). PICRUSt2 for prediction of metagenome functions. Nat Biotechnol.

[25] Kanehisa M, Goto S (2000). KEGG: kyoto encyclopedia of genes and genomes. Nucleic Acids Res.

[26] McMurdie PJ, Holmes S (2013). Phyloseq: an R package for reproducible interactive analysis and graphics of microbiome census data. PLoS ONE.

[27] Cai Z, Lin S, Hu S, Zhao L (2021). Structure and function of oral Microbial Community in Periodontitis based on Integrated Data. Front Cell Infect Microbiol.

[28] Huang N, Shimomura E, Yin G, Tran C, Sato A, Steiner A, Heibeck T, Tam M, Fairman J, Gibson FC (2019). 3rd: immunization with cell-free-generated vaccine protects from Porphyromonas gingivalis-induced alveolar bone loss. J Clin Periodontol.

[29] Karched M, Bhardwaj RG, Asikainen SE (2015). Coaggregation and biofilm growth of Granulicatella spp. with Fusobacterium nucleatum and Aggregatibacter actinomycetemcomitans. BMC Microbiol.

[30] Alvarado A, Behrens W, Josenhans C (2019). Protein activity sensing in Bacteria in regulating metabolism and motility. Front Microbiol.

[31] Zhang M, Wang R, Liao Y, Buijs MJ, Li J (2016). Profiling of oral and nasal microbiome in children with cleft palate. Cleft Palate-Craniofacial J.

[32] McCann KS (2000). The diversity–stability debate. Nature.

[33] Funahashi K, Shiba T, Watanabe T, Muramoto K, Takeuchi Y, Ogawa T, Izumi Y, Sekizaki T, Nakagawa I, Moriyama K (2019). Functional dysbiosis within dental plaque microbiota in cleft lip and palate patients. Prog Orthodont.

[34] Liu L, Zhang Q, Lin J, Ma L, Zhou Z, He X, Jia Y, Chen F (2016). Investigating oral Microbiome profiles in children with cleft lip and palate for prognosis of alveolar bone grafting. PLoS ONE.

[35] Warren DW, Hairfield WM, Dalston ET (1990). The relationship between nasal airway size and nasal-oral breathing in cleft lip and palate. Cleft Palate J.

[36] Hurley E, Barrett MPJ, Kinirons M, Whelton H, Ryan CA, Stanton C, Harris HMB, O’Toole PW (2019). Comparison of the salivary and dentinal microbiome of children with severe-early childhood caries to the salivary microbiome of caries-free children. BMC Oral Health.

[37] Iurovschi R, Joaquim CR, de Faveri M, de Miranda TS, Feres M, de Figueiredo LC (2020). Evaluation of the Microbiological Profile of alveolar residual screws and cleft-adjacent teeth in individuals with complete unilateral fissures. Cleft palate-craniofacial Journal: Official Publication Am Cleft Palate-Craniofacial Association.

[38] Roode GJ, Bütow KW (2018). A descriptive study of Chlorhexidine as a disinfectant in cleft palate surgery. Clin Med Res.

[39] Jousimies-Somer H, Grénman R, Rintala A (1986). Bacteriological investigation of secretory otitis media in children with cleft palate. Scand J Plast Reconstr Surg.

[40] Karched M, Bhardwaj RG, Asikainen SE (2015). Coaggregation and biofilm growth of Granulicatella spp. with Fusobacterium nucleatum and Aggregatibacter actinomycetemcomitans. BMC Microbiol.

[41] Xu L, Zhu Y, Ren L, Xu B, Liu C, Xie Z, Shen K (2017). Characterization of the nasopharyngeal viral microbiome from children with community-acquired pneumonia but negative for Luminex xTAG respiratory viral panel assay detection. J Med Virol.

[42] Oliveira IMFd, Ng DY, van Baarlen P, Stegger M, Andersen PS, Wells JM (2022). Comparative genomics of Rothia species reveals diversity in novel biosynthetic gene clusters and ecological adaptation to different eukaryotic hosts and host niches. Microb Genomics.

[43] Keskitalo A, Munukka E, Aatsinki A, Saleem W, Kartiosuo N, Lahti L, Huovinen P, Elo LL, Pietilä S, Rovio SP et al. An infancy-onset 20-Year dietary counselling intervention and gut microbiota composition in Adulthood. Nutrients 2022, 14(13). 10.3390/nu14132667 PMC9268486 35807848

[44] Nouioui I, Carro L, García-López M, Meier-Kolthoff JP, Woyke T, Kyrpides NC, Pukall R, Klenk HP, Goodfellow M, Göker M. Genome-Based Taxonomic Classification of the Phylum Actinobacteria. *Frontiers in microbiology* 2018, 9:2007. 10.3389/fmicb.2018.02007 PMC6113628 30186281

[45] Pudlo NA, Urs K, Kumar SS, German JB, Mills DA, Martens EC. Symbiotic human gut bacteria with variable metabolic priorities for host mucosal glycans. MBio. 2015;6(6). 10.1128/mbio. 01282 – 01215. 10.1128/mBio.01282-15 PMC4659458 26556271

[46] Brown HA, Marnett LJ. Introduction to lipid biochemistry, metabolism, and signaling. Volume 111. ACS; 2011. pp. 5817–20. 10.1021/cr200363s 21951202

[47] Romine MF, Rodionov DA, Maezato Y, Osterman AL, Nelson WC (2017). Underlying mechanisms for syntrophic metabolism of essential enzyme cofactors in microbial communities. ISME J.

[48] Shames SR, Auweter SD, Finlay BB (2009). Co-evolution and exploitation of host cell signaling pathways by bacterial pathogens. Int J Biochem Cell Biol.

[49] Alvarado A, Behrens W, Josenhans C (2020). Protein activity sensing in bacteria in regulating metabolism and motility. Front Microbiol.

