# Supplementary material for: Investigating oral microbiome profiles in patients with cleft lip and palate compared with the healthy control
Source: BMC Oral Health. 2024 May 28;24:623. doi: 10.1186/s12903-024-04387-3 (PMC11134767; doi:10.1186/s12903-024-04387-3)
Supplement: Supplementary file 1 — Supplementary Material 1 [file 12903_2024_4387_MOESM1_ESM.doc]

Supplementary Method

- DNA extraction

DNA was extracted using a CTAB protocol [1], optimized for high-yield and purity. Initially, 1 ml of 65 °C preheated CTAB extraction solution was added to the sample-containing centrifuge tube, followed by thorough mixing on a vortex mixer to fully suspend the sample. This mixture was then incubated in a 65 °C water bath for 60 minutes, with intermittent shaking 2–3 times to ensure complete lysis, and allowed to cool to room temperature. The lysate was centrifuged at 8000 rpm for 5 minutes, and the supernatant was transferred to a new sterile 2.0 ml centrifuge tube. Chloroform: isoamyl alcohol (24:1) in a volume of 800 µl was added, mixed by inverting 100 times, and centrifuged at 12000 rpm for 20 minutes. This step was repeated with 600 µl of the supernatant in a new tube, followed by the addition of an equal volume of chloroform: isoamyl alcohol (24:1), and centrifugation. Then, 400 µl of the clear supernatant was transferred to a new 1.5 ml sterile tube, mixed with 2/3 volume of isopropanol and 1/10 volume of 3M sodium acetate, incubated at -20 °C for 1 hour, and centrifuged at 12000 rpm for 10 minutes. The supernatant was discarded, and the DNA pellet was dried at room temperature for 10 minutes until semi-translucent. Finally, the pellet was dissolved in 50-100 µl sterile ddH2O with 10 mg/ml DNase, digested at 37 °C for 1 hour, and stored at -20 °C.

*Reference:*

1. *Chen F, Ye J, Chio C, Liu W, Shi J, Qin W: A simplified quick microbial genomic DNA extraction via freeze-thawing cycles. Molecular biology reports 2020, 47(1):703-709.*

Supplementary Table

Table S1 The relative abundance for the top 20 genera.

| Genera | CLP | HC |
| --- | --- | --- |
| *Streptococcus* | 29.61% | 30.27% |
| *Rothia* | 2.43% | 13.53% |
| *Prevotella* | 8.82% | 8.93% |
| *Neisseria* | 18.62% | 5.99% |
| *Veillonella* | 2.10% | 6.85% |
| *Pauljensenia* | 2.07% | 6.26% |
| *Haemophilus* | 9.75% | 4.06% |
| *Porphyromonas* | 5.40% | 2.86% |
| *Actinomyces* | 1.90% | 3.34% |
| *Granulicatella* | 4.63% | 2.33% |
| *Gemella* | 2.67% | 2.72% |
| *Alloprevotella* | 2.41% | 1.44% |
| *Fusobacterium* | 1.72% | 1.54% |
| *Nanosynbacter* | 0.85% | 1.60% |
| *Leptotrichia* | 0.71% | 1.37% |
| *Capnocytophaga* | 1.39% | 0.45% |
| *Lancefieldella* | 0% | 0.82% |
| *Centipeda* | 0.23% | 0.71% |
| *Eubacterium* | 0.31% | 0.61% |
| *Anaeroglobus* | 0.16% | 0.61% |

Supplementary Figure


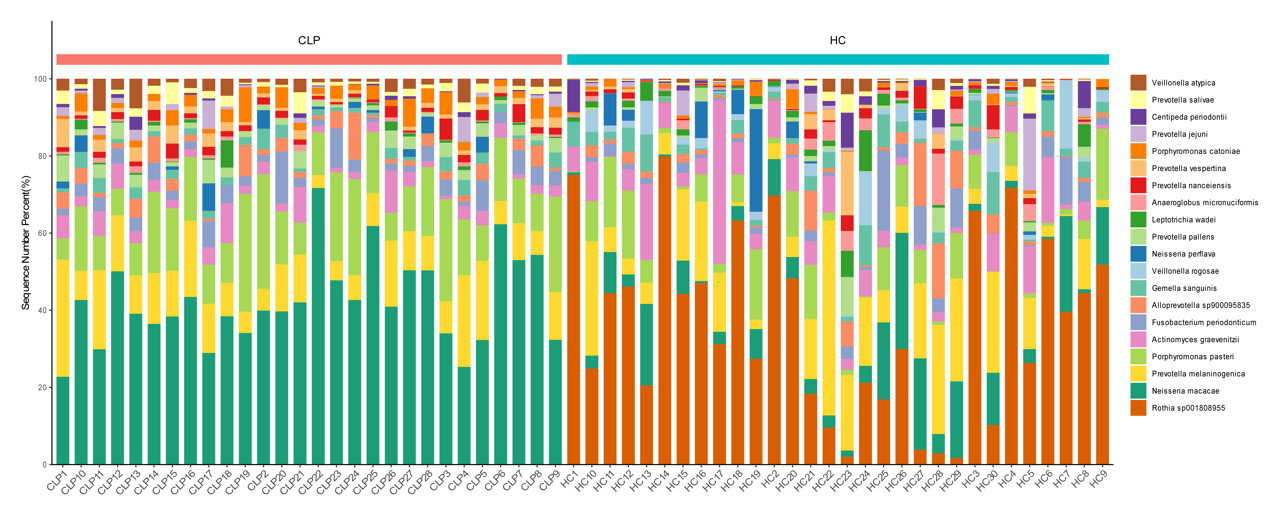


Fig.S1 Microbiological profiles at the species level in the CLP and HC groups. The bar graphs illustrate the mean relative abundances of the top 20 species identified in the study cohorts. CLP: cleft lip and palate; HC: healthy control.
